# Progress on the Cu-Catalyzed 1,4-Conjugate Addition to Thiochromones

**DOI:** 10.3390/catal13040713

**Published:** 2023-04-08

**Authors:** Fenghai Guo, Jayla A. Young, Mina S. Perez, Holden A. Hankerson, Alex M. Chavez

**Affiliations:** 1 Department of Chemistry, Winston-Salem State University, 601 S. Martin Luther King Jr. Dr., Winston-Salem, NC 27110, USA; 2 Biomedical Research Infrastructure Center, Winston-Salem State University, Winston-Salem, NC 27110, USA

**Keywords:** organocopper reagents, Cu catalysts, 1,4-conjugate addition, sulfur heterocycles, thiochromone, thioflavanones, 2-alkylthiochroman-4-ones

## Abstract

Carbon–carbon bond formation is one of the most important tools in synthetic organic chemists’ toolbox. It is a fundamental transformation that allows synthetic chemists to synthesize the carbon framework of complex molecules from inexpensive simple starting materials. Among the many synthetic methodologies developed for the construction of carbon–carbon bonds, organocopper reagents are one of the most reliable organometallic reagents for this purpose. The versatility of organocuprate reagents or the reactions catalyzed by organocopper reagents were demonstrated by their applications in a variety of synthetic transformations including the 1,4-conjugate addition reactions. Sulfur-containing heterocyclic compounds are a much less studied area compared to oxygen-containing heterocycles but have gained more and more attention in recent years due to their rich biological activities and widespread applications in pharmaceuticals, agrochemicals, and material science. This paper will provide a brief review on recent progress on the synthesis of an important class of sulfur-heterocycles-2-alkylthiochroman-4-ones and thioflavanones via the conjugate additions of Grignard reagents to thiochromones catalyzed by copper catalysts. Recent progress on the synthesis of 2-substituted thiochroman-4-ones via alkynylation and alkenylation of thiochromones will also be covered in this review.

## 1. Introduction

Carbon–carbon bond formation is one of the most important tools in synthetic organic chemists’ toolbox. It is a fundamental transformation that allows synthetic chemists to synthesize the carbon framework of complex molecules from inexpensive simple starting materials. Among the many synthetic approaches developed for the construction of carbon–carbon bonds, organocopper reagents are one of the most reliable organometallic reagents for this purpose [1–8]. A wide range of organocopper reagents were developed for carbon–carbon bond formations [2]. Organometallic reagents catalyzed by copper (I) salts or/and organocopper reagents were successfully employed to construct carbon–carbon bonds sp, sp^2^, and sp^3^ carbon centers [2]. The applications of cuprate reagents in a variety of synthetic transformations including the 1,4-conjugate addition reactions further demonstrated their versatility and usefulness [2,3]. Although the conjugate addition of stoichiometric amount of organocuprates to thiochromones were also reported [9,10], this review will focus on recent progress on the conjugate additions of organocopper reagents to thiochromones especially those using catalytic amount of copper (I) salts in the synthesis of an important class of sulfur-heterocycles-2-alkylthiochroman-4-ones and thioflavanones. Other synthetic approaches to 2-alkylthichroman-4-ones and thioflavanones were well summarized in a recent review article [11] and this review aims to provide more details on the progress on synthesis of 2-substituted thichroman-4-ones via conjugate addition of Grignard reagents catalyzed by copper salts. This review also aims to give an introduction to the history, development of the organocuprates and especially their applications in the synthesis 2-substituted thiochroman-4-ones. Progress on the synthesis of 2-substituted thiochroman-4-ones via alkynylation and alkenylation of thiochromones will also be covered in this review as they represent a nice addition to the existing synthetic approaches towards the synthesis of 2-substituted thiochroman-4-ones.

Sulfur-containing heterocycles are important due to their widespread presence in numerous bioactive natural products as well as pharmaceuticals [12–15]. They have widespread applications in many areas including biology, medicinal chemistry, food chemistry, and material science [16–22]. The improvement of the bioavailability and bioactivity was reported by the isosteric replacement of an oxygen atom by a sulfur atom [23], but sulfur-containing heterocyclic compounds are a much less studied area compared to oxygen-containing heterocycles. Due to their widespread applications and rich biological activities, the development towards an efficient synthetic approach to sulfur-containing compounds received more and more interest from both industry and academia. This paper will provide a brief review on recent progress on the synthesis of an important class of sulfur-heterocycles-2-alkylthiochroman-4-ones and thioflavanones via the conjugate additions of Grignard reagents to thiochromones catalyzed by copper catalysts. Recent reports on the synthesis of 2-alkynyl thiochroman-4-ones and 2-alkenylation thiochromones will also be covered in this review. This review aims to cover recent progress on the addition of alkyl, aryl, alkenyl, to thiochromones via 1,4-conjugate addition of Grignard reagents as well as the formal conjugate addition of alkynyl groups to thiochromones catalyzed of Cu (I) salts.

### 1.1. Introduction to the Pioneering Studies on Organocopper Reagents

**Figure 1.**
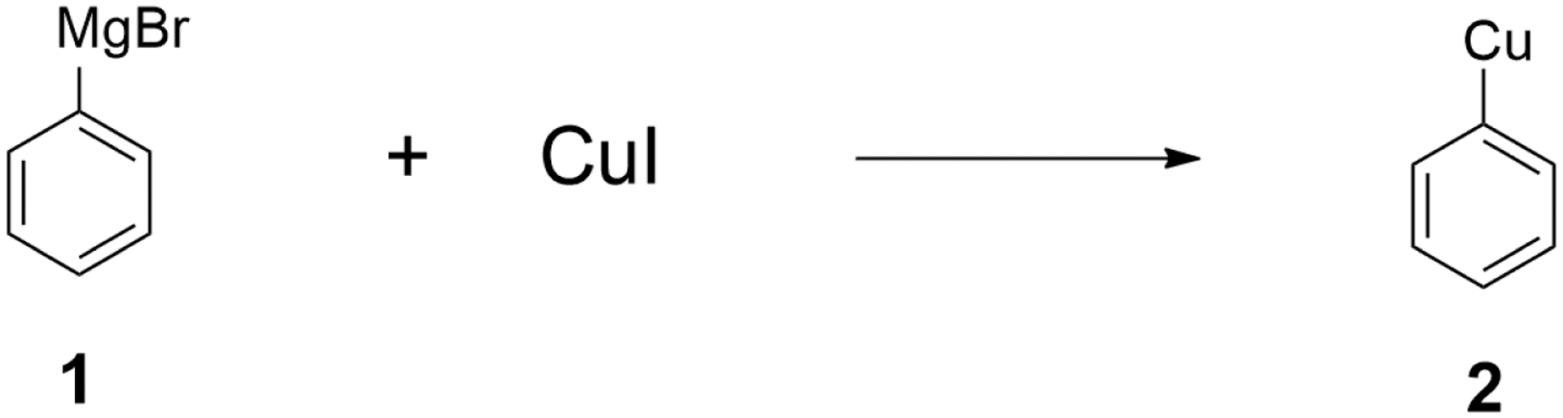
Phenyl copper prepared from phenyl magnesium bromide.

Among the many synthetic approaches developed for the construction of carbon–carbon bonds, organocopper reagents are one of the most reliable organometallic reagents for this purpose [1–8]. Among the earliest examples of organocopper chemistry is Glaser’s employment of alkynylcopper reagents in the synthesis of diynes from terminal alkynes in 1870 [24]. Other pioneering studies on organocopper chemistry include the copper-catalyzed Ullmann biaryl synthesis [25] and aryl ether synthesis [26]. Only after more than 50 years later, in 1923, Reich successfully used copper (I) Iodide and phenyl magnesium bromide (**1**) to prepare phenyl copper [27] (i.e., **2**, Figure 1). This was groundbreaking in terms of the development of organocopper reagents, as this was the first preparation of a stoichiometric organocopper reagent and, thus, it marked the beginning of organocopper chemistry.

**Figure 2.**
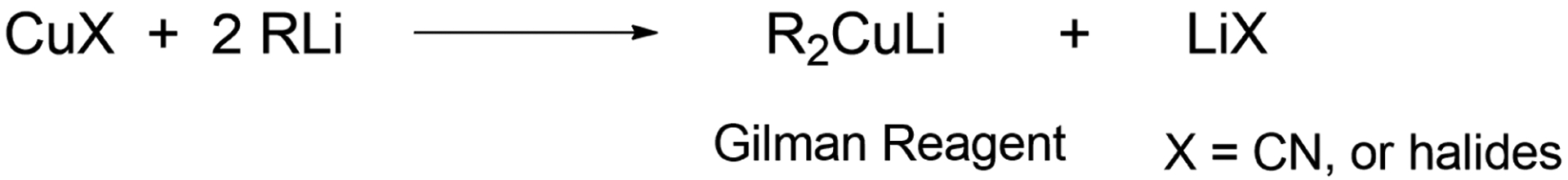
The First Dialkylcuprate Reagent Prepared by Gilman.

Since the groundbreaking preparation of phenyl copper, much progress in the field of organocopper reagents was reported. For example, more than a decade later, Gilman and Straley demonstrated the synthetic potential of a mono-organocopper reagent when they successfully prepared the first mono-alkylcopper reagent (i.e., ethylcopper) from copper (I) iodide (CuI) and ethyl magnesium iodide (EtMgI) [28] in 1936. Five years later, Karasch [29] discovered the 1,4-conjugate addition of Grignard reagents to α, β-unsaturated ketones catalyzed by copper (I) chloride. This is a very important discovery as it showed that copper (I) salts such as CuCl can selectively affect the 1,4-conjugate addition of Grignard reagents to α, β-unsaturated ketones over 1,2-additon to carbonyl group. Later, the first organocuprate reagent, lithium dimethylcuprate (Me_2_CuLi), was prepared and used in the acylation reactions for ketone synthesis by Gilman in 1952 [30]. These lithium dialkylcuprates were later named “Gilman” reagents in honor of Gilman for his pioneering contributions in this area (Figure 2).

Stoichiometric lithium dialkylcuprates (i.e., R_2_CuLi) were later used by House and Whitesides to effect 1,4-conjugate addition to α, β-enones [31]. This reaction was highly chemoselective (i.e., 1,4-adduct vs. 1,2-adduct), affording an excellent chemical yield (1,4-adduct vs. 1,2-adduct) and high reproducibility [31]. Thus, this reaction by House and coworkers further demonstrated that cuprates are the reactive species in Karasch’s CuCl catalyzed 1,4-addition of MeMgBr to α,β-enones. Although the reactive species was not clear at that time, Karsch observed that Grignard reagents underwent 1,4-conjugate addition to α,β-enones instead of 1,2-addition to afford the 1,4-adducts selectively in the presence of only 1% copper (I) salt [29]. Grignard reagents usually underwent the 1,2-addition pathway over 1,4-additon without addition of copper (I) salt. This pioneering discovery opens the door for many synthetic applications of copper-catalyzed Grignard reagents. Although there are problems of reproducibility, copper-catalyzed Grignard reactions are highly synthetically useful due to the readily availability from commercial sources as well as the easy preparation of Grignard reagents from corresponding halides. Later, the reproducibility problem can be solved by using highly pure, and stable copper salts such as CuBr●SMe_2_ and CuCN that are soluble. The utility of copper-catalyzed Grignard reagents increases with the reproducibility problem minimized. One of the drawbacks of organocuprates is they usually require the stoichiometric amount of Cu (I) salts. Using excess copper (I) salt is always less appealing from an environmental consideration. If problems such as low reproducibility and the competing side reactions (i.e., competition of 1,2-addition by orgnolithium and Grignard reagents) can be solved, it is always more appealing to develop and use procedures that only require catalytic amount of copper (I) salt than stoichiometric amount of organocuprate reagents. This paper will provide a brief review on recent progress on the conjugate additions of Grignard reagents to thiochromones catalyzed by copper catalysts as they provide a quick entry into an important class of sulfur-heterocycles-2-alkylthiochroman-4-ones and thioflavanones. The progress on the 1,4-additions of alkenyl Grignard reagents promoted by TMSOTf and the formal conjugate addition of alkynyl groups to thiochromones catalyzed by Cu (I) salts will also be covered as they represent nice addition to the existing synthetic approaches to 2-substituted thiochroman-4-ones.

Organocopper reagents can be readily prepared from a copper (I) salt and the corresponding Grignard reagents or organolithium reagents. This can be carried out by adding one equivalent of organolithium reagent (RLi) or Grignard reagents (RMgX) to a copper (I) salt. Organocopper reagents could be very efficient reagents based on the organic group transferred, as only one valuable ligand is required here. However, the synthetic applications of this reagent were very limited at the early stage of discovery because of the insoluble nature of some organocopper reagents and, thus, lack of reactivity. For example, methyl copper is a yellow polymeric precipitate [32] in diethyl ether and, as a result, not very reactive, which limited its synthetic applications. Recent advances in combining Lewis acids, such as TMSCl or BF_3_●OEt_2_, with organocuprates such as alkylcopper reagents led to improved reactivity of these reagents and, thus, made them more useful tools for modern organic synthesis [2].

Although the conjugate addition of stoichiometric amount of organocuprates to thiochromones were also reported, [9,10] this paper will focus on recent progress on the conjugate additions of organocopper reagents to thiochromones, especially those using a catalytic amount of copper (I) salts in the synthesis of an important class of sulfur-heterocycles-2-alkylthiochroman-4-ones and thioflavanones. The ability to add alkenyl and alkynyl groups are highly desirable in organic synthesis. This review will also cover the recent progress on the synthesis of 2-alkynyl thiochroman-4-ones via the Cu-catalyzed formal conjugate addition of alkynyl groups to thiochromones and 2-alkenylation thiochromones via 1,4-additions of alkenyl Grignard reagents promoted by TMSOTf.

### 1.2. Significance of Sulfur-Containing Heterocycles-Thiochromanone Derivatives

Due to their wide presence in numerous pharmaceutical active molecules as well as in many bioactive natural products [12–15], sulfur-containing heterocycles played a very important role in our daily lives. Sulfur-containing heterocycles are also widely used in areas, such as medicinal chemistry, material science, food industry, and biology in recent years [16–22]. Although improved bioavailability and bioactivity of compounds are expected via the isosteric replacement of an oxygen atom by a sulfur atom [23], heterocyclic compounds contained sulfur are a much less studied area compared to heterocycles contained oxygen. The development of efficient synthetic approaches to sulfur-containing compounds received more and more interest both in industry and academia in recent years. This is mostly due to the rich biological activities that sulfur-containing compounds display as well as their widespread applications in many areas including pharmaceutical, agrochemical, biology, food chemistry, and other areas. Sulfur-containing heterocycles were found to display rich biological activities. For example, they were found to display cytotoxic effects on tumor cells in vitro [33], cytotoxic activities and the in vitro antileishmanial [34]. Sulfur heterocycles were also reported to be able to kill tumor cells by inducing tumor cell apoptosis [35]. The sulfur analogues of flavonoids, i.e., thioflavonoids, [36–39], were reported to possess rich biological activities. They were reported to have the ability to inhibit nitric oxide production, and to display antimicrobial, antifungal, and antioxidant properties, etc. [40–48]. Thiochromanones, i.e., thiochroman-4-ones, 2-substituted thiochroman-4-ones such as 2-alkylthiochroman-4-ones, and 2-arylthiochroman-4-ones (thioflavanone), are vital precursors and valuable synthons for the synthesis of thiochromanone derivatives for the study of biological activities [49–56].

## 2. Cu-Catalyzed Conjugate Addition of Grignard Reagents to Thiochromones

Carbon–carbon bond formation is one of the most important transformations in the synthesis of carbon framework of complex molecules in organic synthesis. Carbon–carbon bond formation is one of the most important tools in a synthetic organic chemist’s toolbox.

**Figure 3.**
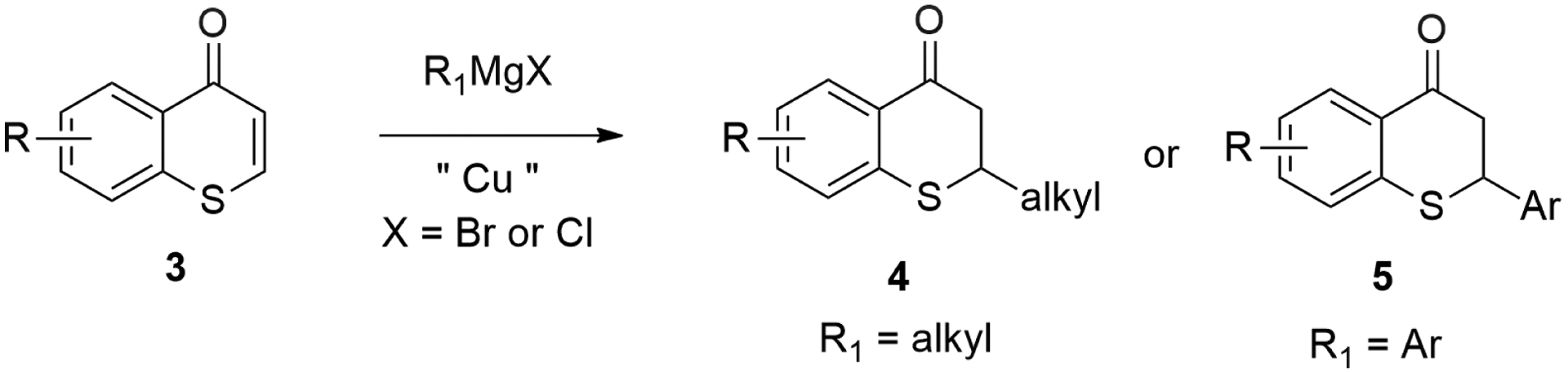
Copper-Catalyzed Conjugate Addition of Grignard Reagents to Thiochromones.

Among the many synthetic approaches developed for the efficient construction of carbon–carbon bonds, the 1,4-conjugate addition reaction of various organometallic reagents, including Grignard reagents (RMgX, X = Br, Cl, I), represented one of the most reliable synthetic methods for this purpose in organic synthesis [57]. It is well known that Grignard reagents usually undergo 1,2-addition to α, β-unsaturated carbonyl compounds selectively over 1,4-addition pathway without the addition of catalysts, such as Cu (I) salts. It was reported that Cu (I) salts effectively catalyzed the 1,4-conjugate addition of Grignard reagents to carbonyl compounds [29,57]. This kind of Cu (I) salt catalyzed reaction of Grignard reagents usually gave excellent regioselectivity (i.e., 1,4-addition vs. 1,2-additon pathway) as exclusive 1,4-adducts were observed [29,57]. Recently, a unified approach to 2-substituted thiochroman-4-ones-2-alkyl thiochroman-4-ones, 2-aryl thiochroman-4-ones (thioflavanones) via Cu-catalyzed 1,4-conjugate addition of Grignard reagents to thiochromones was reported by our research group (Figure 3) [58]. This unified approach took advantage of the readily available of Grignard reagents, the ease in preparation from corresponding halide compounds, and the broad scope of Grignard reagents (Figure 3) [58].

**Figure 4.**
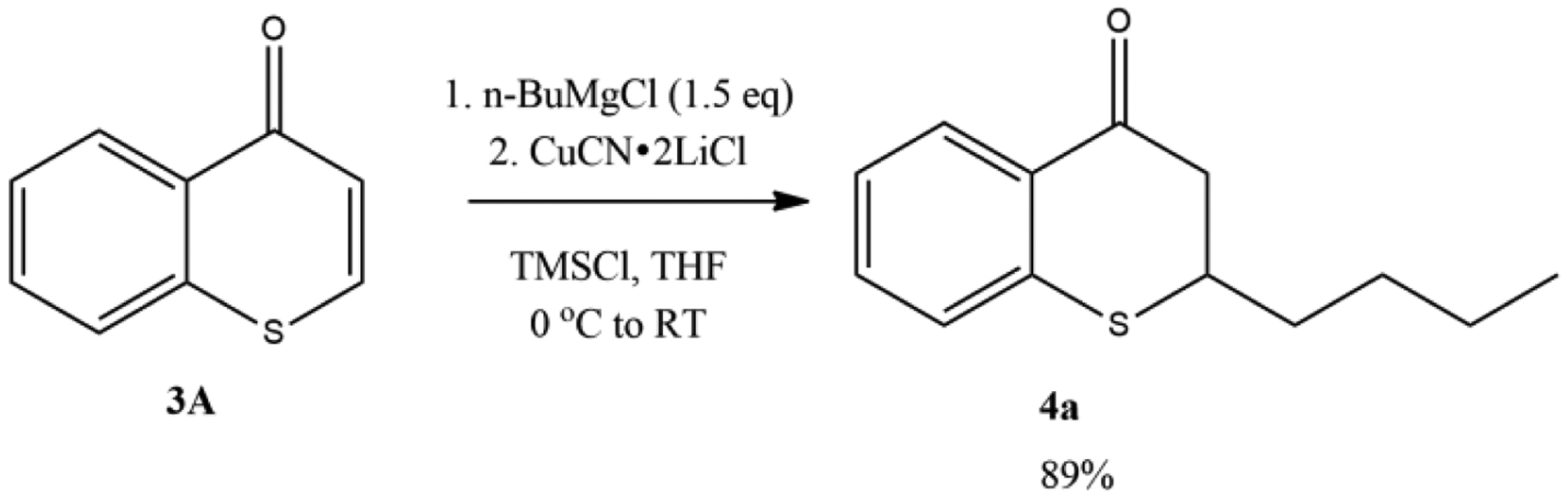
Copper-Catalyzed Conjugate Addition of *n*-BuMgCl to Thiochromones.

The investigation started with *n*-butylmagnesium chloride with thiochromone **3A** (Figure 4). It was reported that an excellent yield (89%) of 1,4-adduct, 2-*n*-butylthiochroman-4-one **4a** can be attained when TMSCl was used as the additive with 2.0 equivalent of LiCl and 1.0 equivalent of CuCN (Figure 4). The optimization of reaction conditions found that the use of 0.2 equivalent of CuCN·2LiCl offered the highest yield of 1,4-adduct with TMSCl as the additive. When a smaller amount of CuCN·2LiCl (0.1 equivalent) was used, no significant change of the yield of 1,4-adduct **4a** (85% vs. 89%) was observed [58].

**Figure 5.**
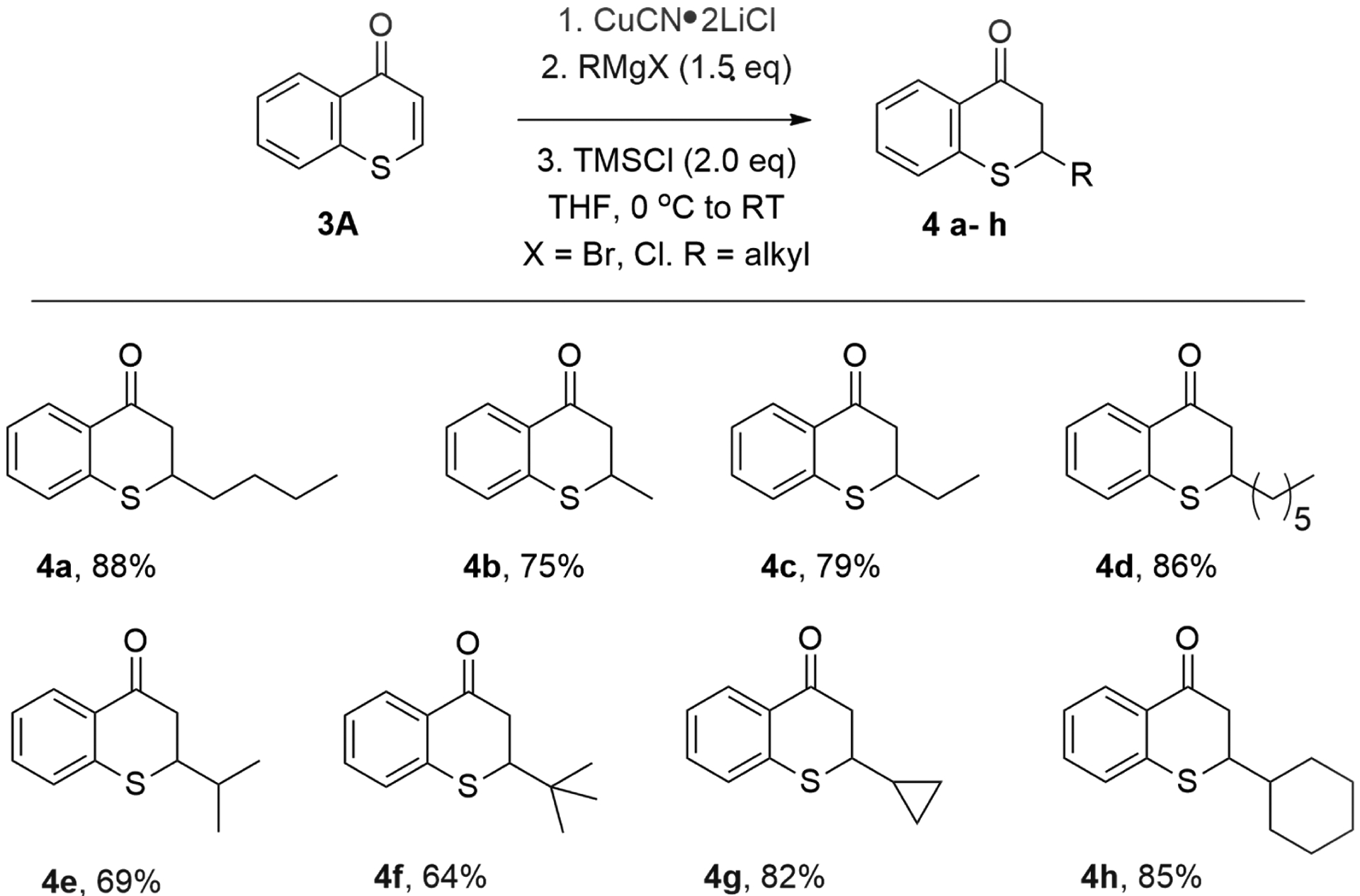
Copper-Catalyzed Conjugate Addition of RMgX to Thiochromones.

With this optimal reaction condition found, the scope of Grignard reagents was then investigated. A broad scope of Grignard reagents added to thiochromone **3A** to in 1,4-additon fashion to afford 1,4-adducts **4a**–**h** with good chemical yields (Figure 5, 64–88%). Simple Grignard reagents underwent 1,4-conjugate addition to thiochromone **3A** smoothly to deliver 1,4-adducts **4a**–**4d** with excellent efficiency (Figure 5, 75–88%). Steric bulkier Grignard reagents were less reactive and afford lower yields presumably due to steric hindrance (Figure 5, **4e**, 69% and **4f**, 64%). Grignard reagents prepared from corresponding cyclic halides also worked well (Figure 5, **4g**, 82% and **4h**, 85%) [58].

**Figure 6.**
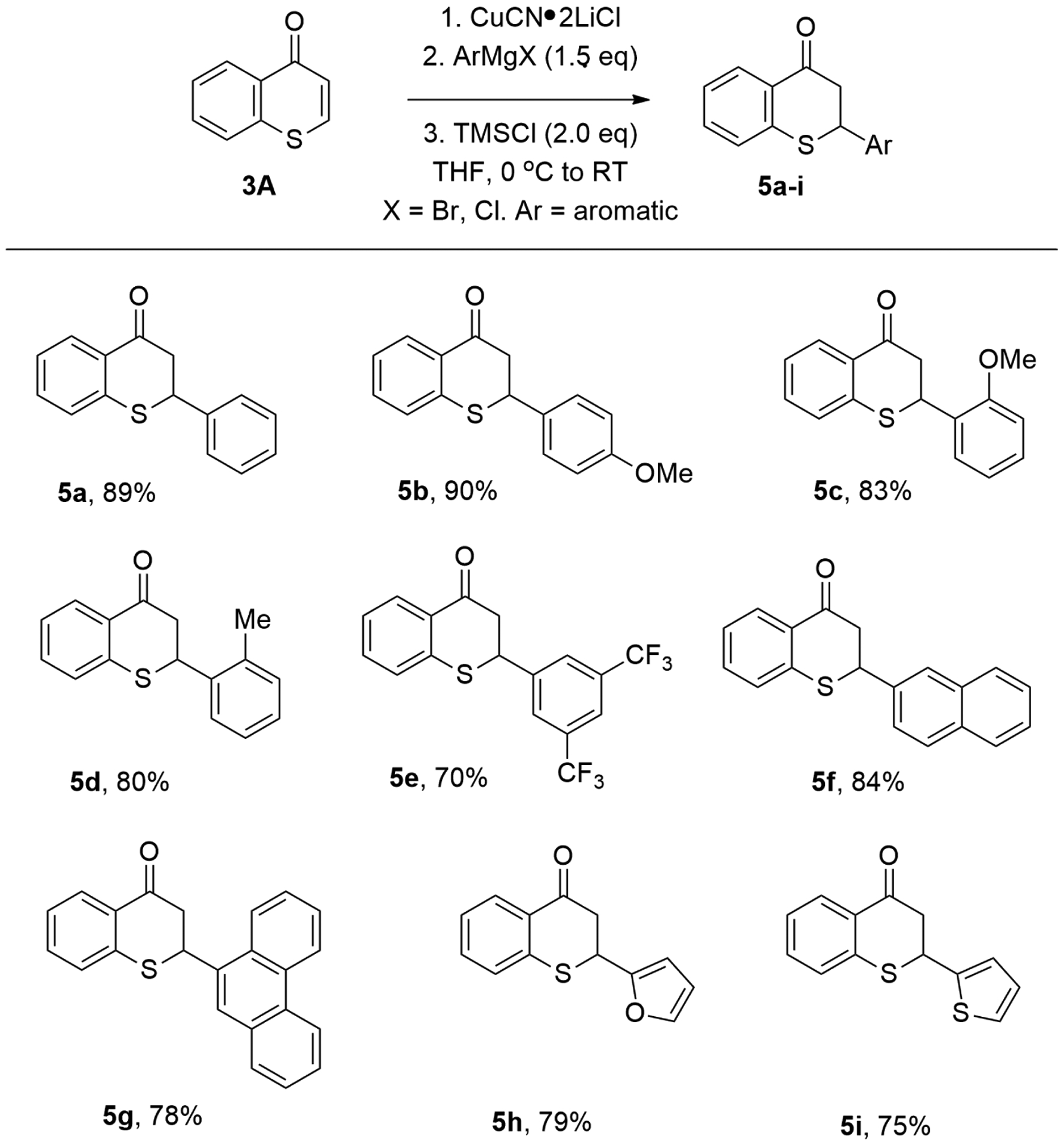
Copper-Catalyzed Conjugate Addition of ArMgX to Thiochromones.

Under these optimal reaction conditions, aromatic Grignard reagents also underwent conjugate addition to thiochromones smoothly (Figure 6). PhMgBr added to thiochromone **3A** to in 1,4-additon fashion with high efficiency (Figure 6, **5a**, 89%). Grignard reagents with electron-donating groups such as “-Me”, “-OMe” on the aromatic ring were found to be effective too (**5b**–**d**, 80–90%). Grignard reagents with strong electron-withdrawing groups such as “-CF_3_” on the aromatic ring also worked well in 1,4-addition fashion (**5e**, 70%). Grignard reagents prepared from extended aryl bromides also underwent 1,4-conjugate addition to thiochromone **3A** smoothly to afford 1,4-adducts with high chemical yields (**5f**, 84%; **5g**, 78%). Furthermore, Grignard reagents prepared from corresponding aromatic heterocycle halides such as 2-furyl)magnesium bromide and 2-thienylmagnesium bromide were also tolerated (**5h**, 79%; **5i**, 75%, Figure 6) [58].

**Figure 7.**
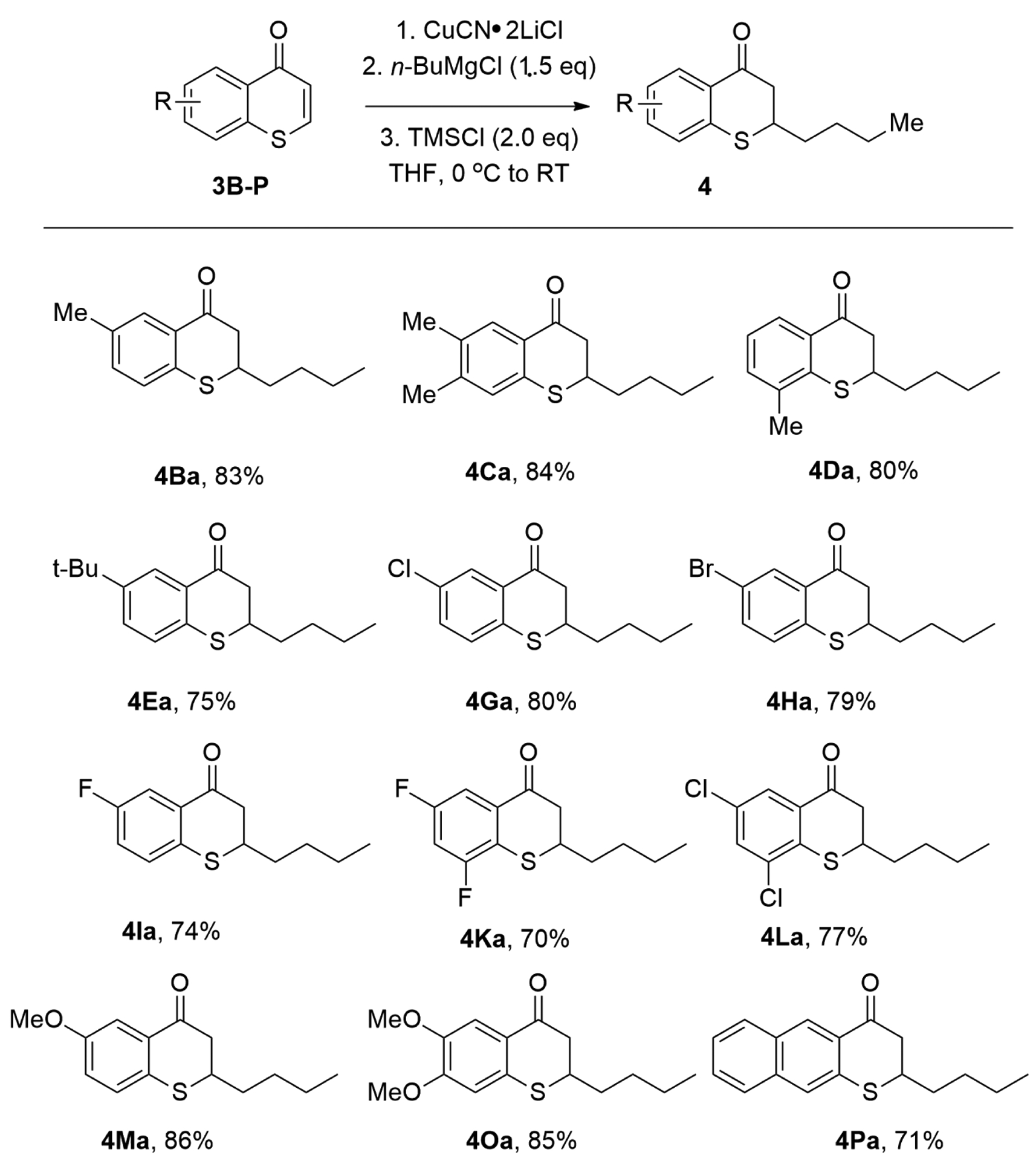
Copper-Catalyzed Conjugate Addition of ArMgX to Thiochromones.

A broad scope of thiochromones worked well under the reported optimal reaction condition (Figure 7). It was reported that *n*-BuMgCl underwent 1,4-conjugate addition to thiochromones **3B**–**3P** smoothly (Figure 7, **4Ba**–**4Oa**, 73–86%). Thiochromones bearing simple alkyl groups reacted well to afford **4Ba**–**4Da** in good yields (80–84%). The steric hindered *t*-butyl group were also tolerated to afford 1,4-adduct **4Ea** with 75% yield. *N*-BuMgCl also added to thiochromones bearing halides on the aromatic ring (i.e., F, Br, and Cl) to deliver 1,4-adducts in good yields (Figure 7). *N*-BuMgCl also underwent 1,4-conjugate addition with thiochromones with two halides on the aromatic ring, such as 6,8-difluorothiochromanone and 6,8-dichlorothiochromone with good efficiency (**4Ka**, 73% and **4La**, 77%, Figure 7). Thiochromones bearing electron-donating groups, such as MeO-, also worked well (Figure 7, **4Ma**, 86%; **4Oa**, 85%). Extended thiochromone **3P** also worked well (Figure 7, **4Pa**, 71%) [58].

**Figure 8.**
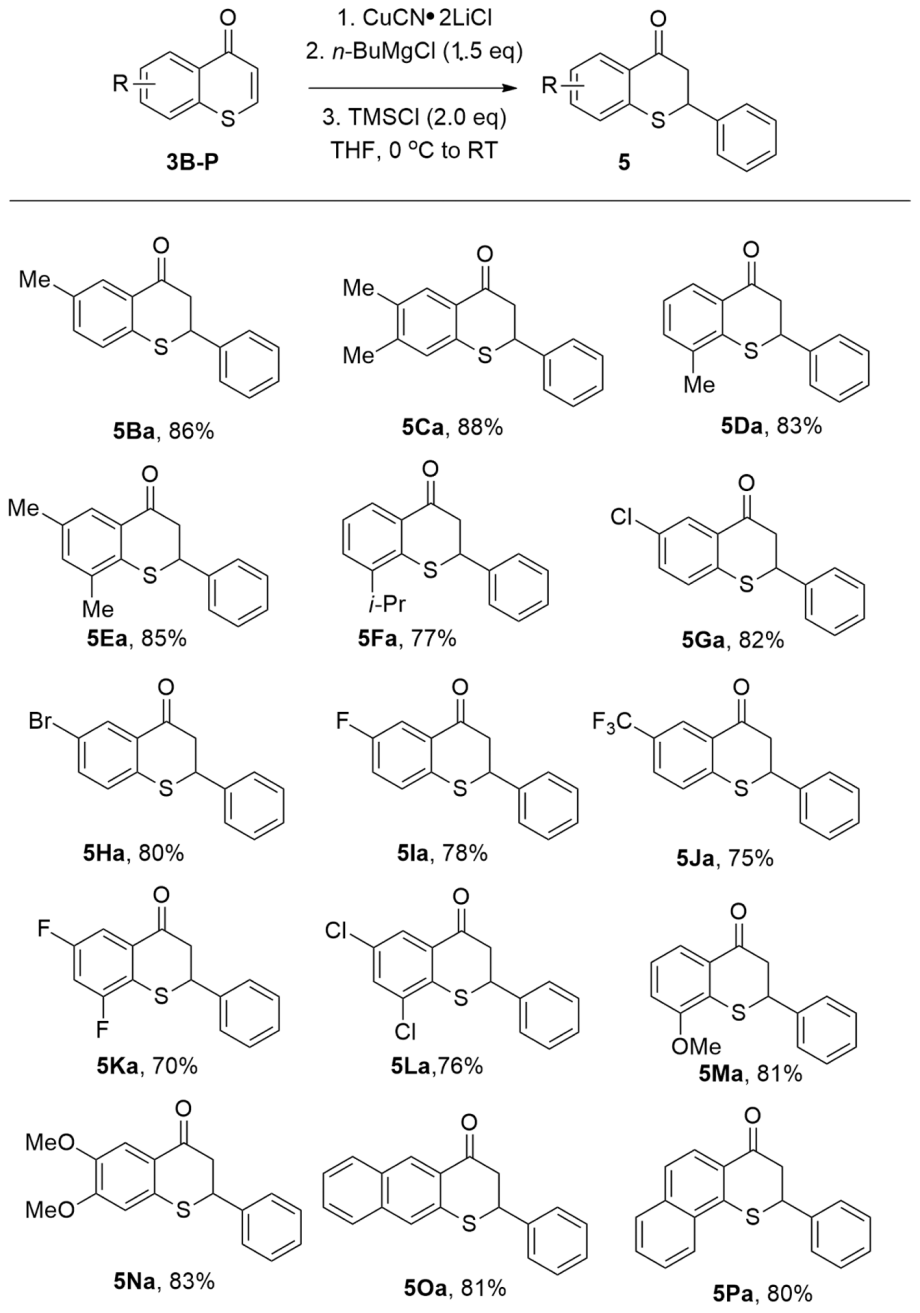
Synthesis of thioflavanones.

With the employment of PhMgBr, the scope of substituted thiochromones was also explored to synthesize various thioflavanones, an important class of thiochromone derivatives with rich biological activities. Thiochromones bearing electron-donating or electron-withdrawing groups on the aromatic ring were investigated with PhMgBr. PhMgBr added to thiochromones **3B**–**3P** in 1,4-additon fashion smoothly to afford 1,4-adducts-thioflavanones in good yields (Figure 8). For example, with simple alkyl groups on the aromatic ring of thiochromones, thioflavanones **5Ba**–**3Ea** can be isolated in good yields (Figure 8, 83–88%). Thiochromone bearing bulky groups (*i*-Pr) were also tolerated (Figure 8, **5Fa**, 77%). PhMgBr also worked well with thiochromones bearing halides (i.e., F, Br, and Cl) on the aromatic ring (Figure 8, **5Ga**–**5Ia**, 78–82%). PhMgBr also underwent 1,4-conjugate addition with thiochromones with two halides on the aromatic ring, such as 6,8-difluorothiochromanone and 6,8-dichlorothiochromone with good efficiency (Figure 8, **5Ka**, 70% and **5La**, 76%). Electron-donating groups, such as MeO-, also underwent smooth 1,4-conjugate addition to thiochromones to deliver 1,4-adducts in excellent chemical yields (5Ma, 84% and 5Na, 83%). PhMgBr also adds to 8-substituted thiochromones (8-*i*-Pr, 8-MeO-) with good yields (**5Fa**, 77%, **5Ma**, 81%). This indicates that the steric hindrance was not a problem here. Thiochromones with extended aromatic structures also underwent 1,4-conjugate addition with PhMgBr with high efficiency (Figure 8, **5Oa**, 81%; **5Pa**, 80%) [58].

## 3. Enantioselective Cu-Catalyzed Conjugate Addition of Grignard Reagents to Thiochromones

**Figure 9.**
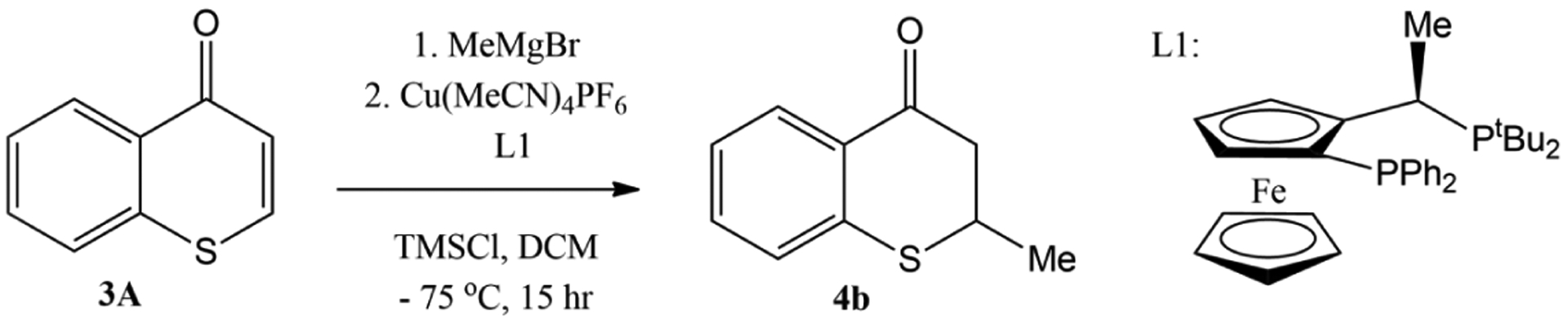
Enantioselective Cu-catalyzed conjugate addition of MeMgBr to thiochromones.

Recently, an enantioselective Cu-catalyzed conjugate addition of Grignard reagents to thiochromones was also reported [59]. The screening of the reaction conditions found that a combination of copper salt, Cu[MeCN]_4_PF_6_, and chiral ligand, (*R*, *S*)-PPF-P^t^Bu_2_, in DCM with TMSCl as the additive offered the best chemical yield (73%) as well as the best enantioselectivity (82% ee) of 1,4-adduct 4b (Figure 9).

**Figure 10.**
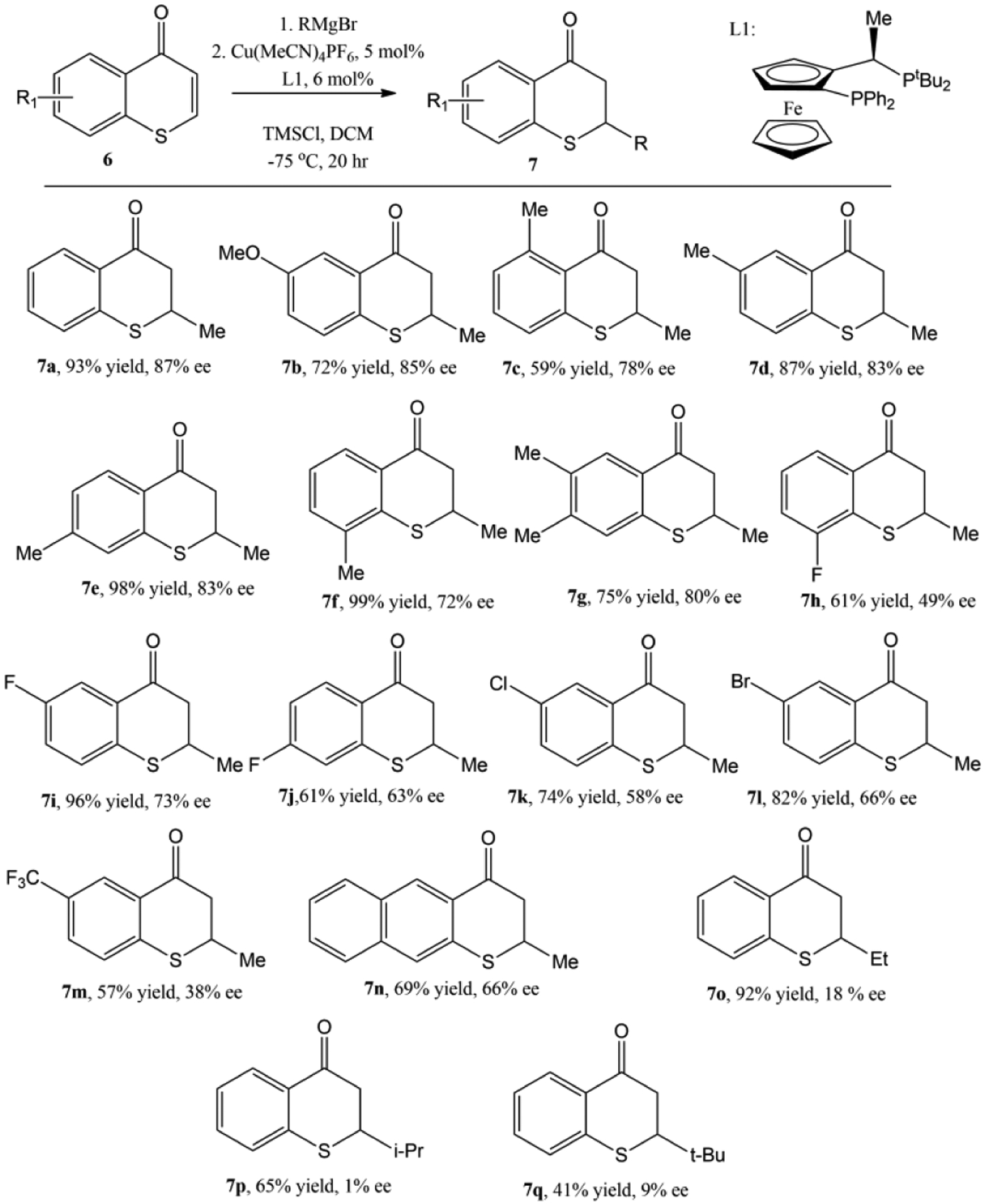
The Scope of Enantioselective Cu-catalyzed conjugate addition to thiochromones.

The optimal reaction condition was ultimately determined to be 0.2 mmol thiochromones in 4 mL DCM with 5 mol% Cu[MeCN]_4_PF_6_ and 6 mol% (*R*, *S*)-PPF-P^t^Bu_2_ at −75 °C using TMSI as the additive [59]. With this optimal reaction in hand, both the scopes of thiochromones and Grignard reagents were explored. Thiochromones with electron donating groups such as MeO or Me undergo conjugate addition with MeMgBr smoothly to afford 1,4-adducts **7a**–**g** in good to moderate yields and enantioselectivites (Figure 10, 59–99% yields, and 72–87% ee). Lower enantioselectivies were observed with thiochromones with F, Cl, Br, CF_3_ groups (**7h**–**m**, 38–73% ee, Figure 10). Thiochromones with extended aromatic group also reacted with MeMgBr to afford 1,4 adduct **7n** in 69% yield and 66% ee (Figure 10). Alkyl Grignard reagents except MeMgBr gave modest to good yields (41–92%) but with poor enantioselectivities (1–18% ee) [59].

## 4. Alkynylation and Alkenylation of Thiochromones

### 4.1. Enantioselective Cu-Catalyzed Alkynylation of Thiochromones

**Figure 11.**
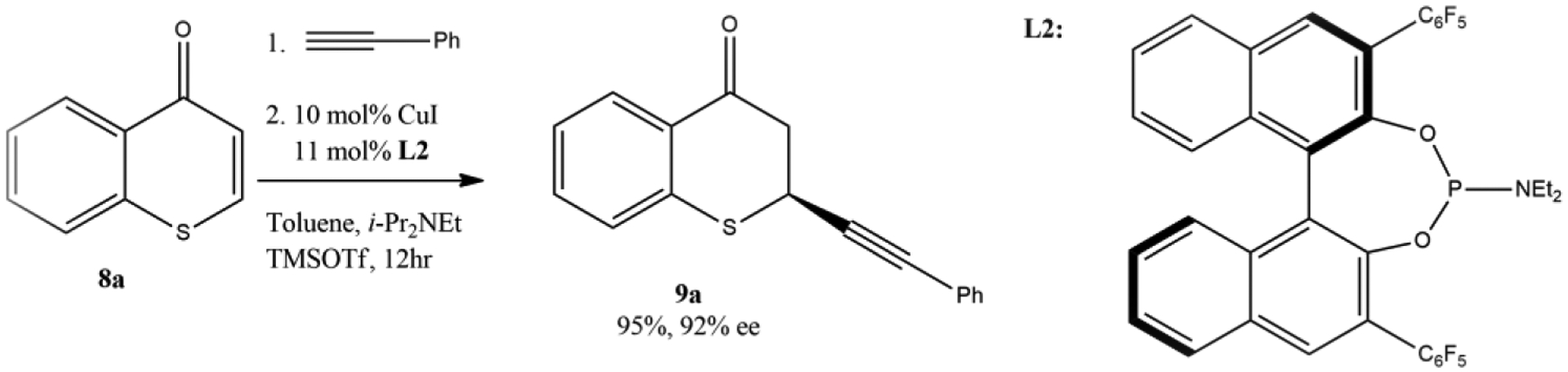
Enantioselective Cu-catalyzed alkynylation of thiochromones.

Cu-catalyzed alkynylation of thiochromones was also reported recently by Wang’s research group in 2020 [60]. The ability to incorporate the alkynyl functional group is important for research in pharmaceuticals and agrochemicals, as well as for functionalized materials. This method provided a unique approach by introducing alkyne functional groups onto thiochromanones, which will offer new opportunities for studying biological activities and further applications in materials science and other fields for these sulfur-containing heterocycles. It was found that the best yield (95%) and enantioselectivity (92% ee) of 1,4-adduct **9a** can be attained with thiochromone **8a** and phenylacetylene when more hindered phosphoramidite ligand **L2** was used (Figure 11). It was found that the additive TMSOTf is important for the success of this alkynylation reaction, as it activates the thiochromone substrates to form corresponding 4-[(trimethylsilyl)oxy]thiochromenylium as key intermediate for this reaction. TMSOTf also serves as a counteranion to stabilize the reactive complex as it was reported that a remarkable anion effect from trimehylsilyl salt was observed and the highest reactivity and enantioselectivity were achieved with the less coordinating triflate (OTf) group [60].

**Figure 12.**
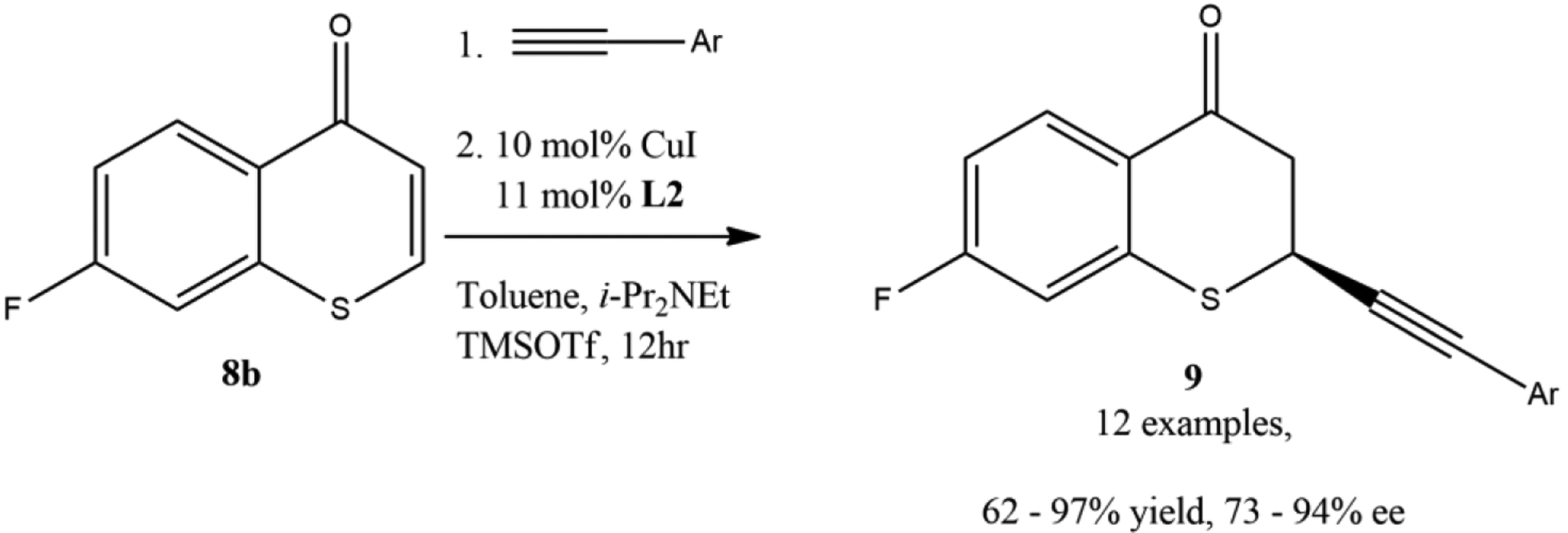
The scope of arylacetylenes for the enantioselective Cu-catalyzed alkynylation of thiochromones.

The scope of arylacetylene was investigated with thiochromone **8b** (Figure 12) and it was found that arylacetylenes bearing both electron-withdrawing and electron-donating groups on the aromatic ring were tolerated to furnish 1,4-adducts **9** in good to excellent yields and enantioselectivities (12 examples, 62–97% yield, 73–94% ee, Figure 12). Additionally, the scope of substituted thiochromones were also investigated. It was reported that both electron-withdrawing and electron-donating groups on the aromatic ring of the substituted thiochromones worked well. However, no significant difference on the reactivity and enantioselectivity between the electron-withdrawing and electron-donating groups on the aromatic ring of the substituted thiochromones were observed [60].

### 4.2. Alkenylation of Thiochromones with Alkenyl Grignard Reagents

**Figure 13.**
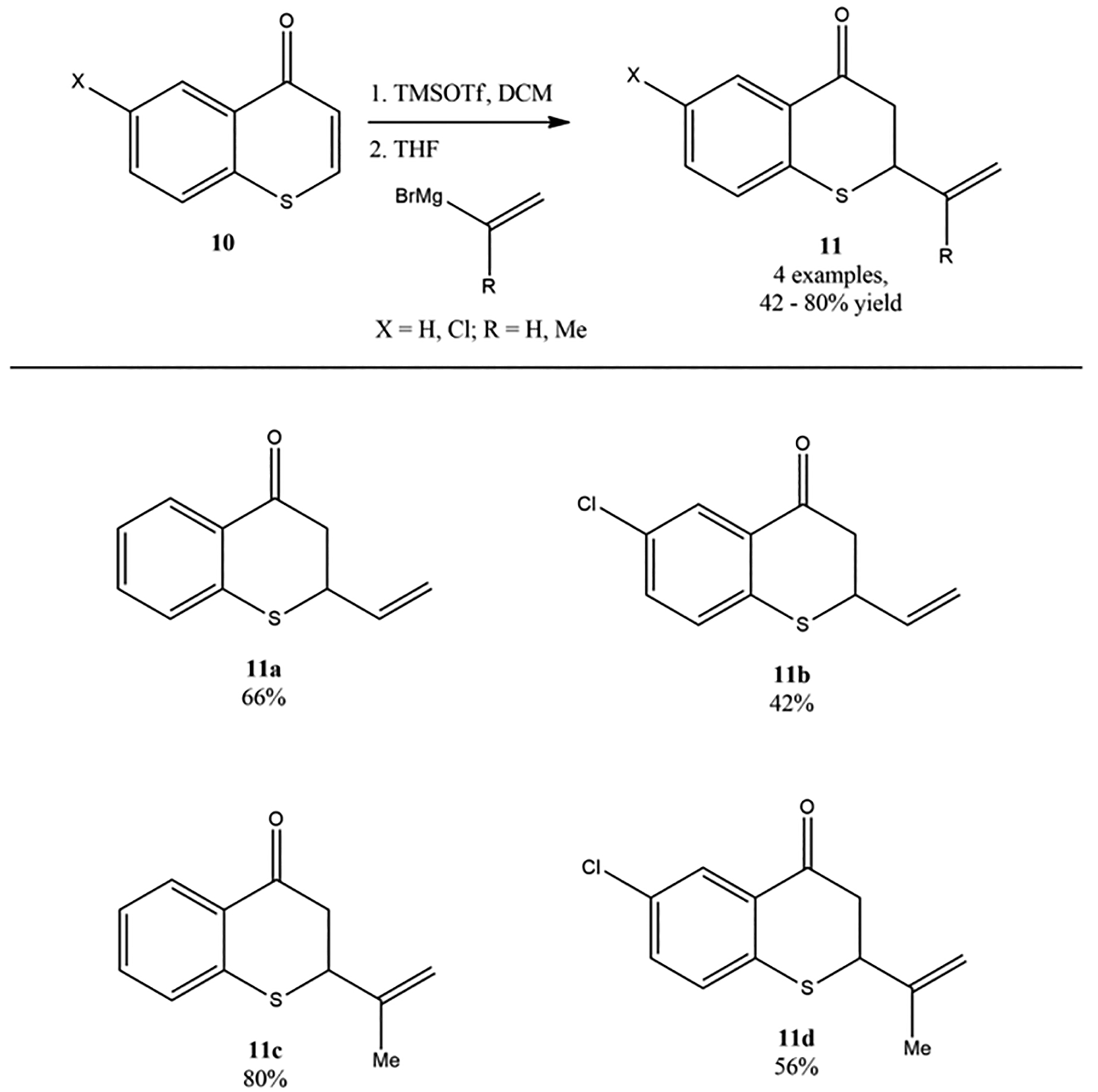
Alkenylation of thiochromones with alkenyl Grignard reagents.

Langer and coworkers reported the alkenylation of thiochromones and chromones with alkenyl Grignard reagents [61]. In their study on the structure–activity relationship of 2-vinylchroman-4-one, 2-vinylchroman-4-ones, and related analogs, the synthesis of 2-alkenylthiochroman-4-ones via the TMSOTf-mediated reaction of thiochromones with vinylmagnesium bromide and 2-(isopropenyl) magnesium bromide were successfully developed (Figure 13). It was noted that the presence of TMSOTf was the key to the success of this reaction. It was mentioned that without the addition of TMSOTf, the reaction of vinylmagnesium bromide and chromone led to complex mixture of compounds due to the competing 1,2-addition to carbonyl group instead of the desired 1,4-addition. The direct addition of alkenyl Grignard reagents to thiochromone and derivatives in the presence of TMSOTf provided a direct synthetic approach to 2-alkenylthiochroman-4-ones by introducing alkene including vinyl functional groups onto thiochromanones. It was found that both vinyl magnesium bromide and 2-(isopropenyl) magnesium bromide worked well with thiochromone to afford 1,4-adducts in modest to good yields (Figure 13). For example, vinyl magnesium bromide added to thiochromone and 6-chlorothichromone in the presence of TMSOTf to deliver 1,4-adducts-2-vinylthiochroman-4-one **11a** (66%) and 6-chloro-2-vinylthiochroman-4-one **11b** (42%). However, 2-(isopropenyl) magnesium bromide also worked well with both thiochromone and 6-chlorothichromone under TMSOTf-mediated reaction condition (Figure 13) [61].

## 5. Mechanism on the Cu-Catalyzed Conjugate Addition of Grignard Reagents to Thiochromones

**Figure 14.**
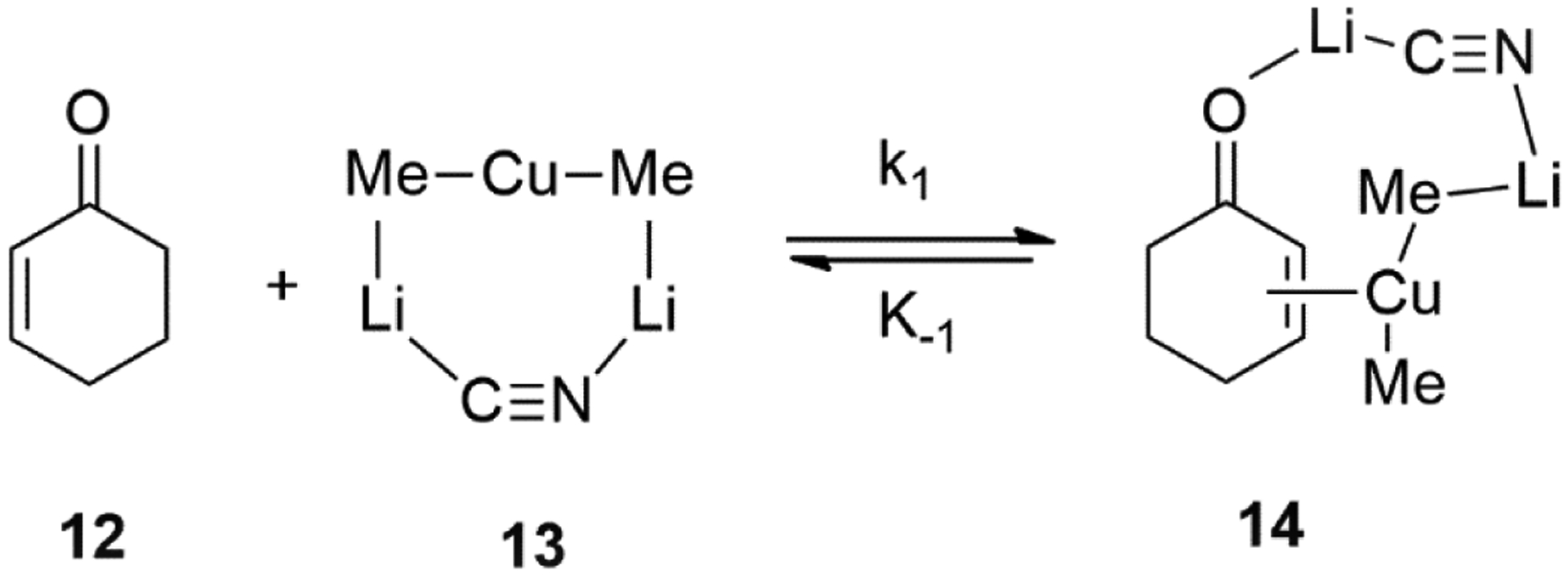
Investigation on Mechanism of Cu-catalyzed conjugate addition of Organocuprates using Rapid-injection NMR techniques.

The mechanism on the 1,4-conjugate addition reactions of cuprate reagents [i.e., Me_2_CuLi●LiCN and Me_2_CuLi●LiI] with 2-cyclohexen-1-one **12** in THF were investigated by Bertz and Ogle [62]. Different π-complexes (i.e., **14**) of cuprate reagents with 2-cyclohexen-1-one was identified in THF using rapid-injection NMR techniques (Figure 14). It was assumed that the contact ion-pairs (CIPs) are the reactive species and, thus, a heterodimeric structure of the contact ion-pair (CIP) **13** in THF was proposed [62].

**Figure 15.**
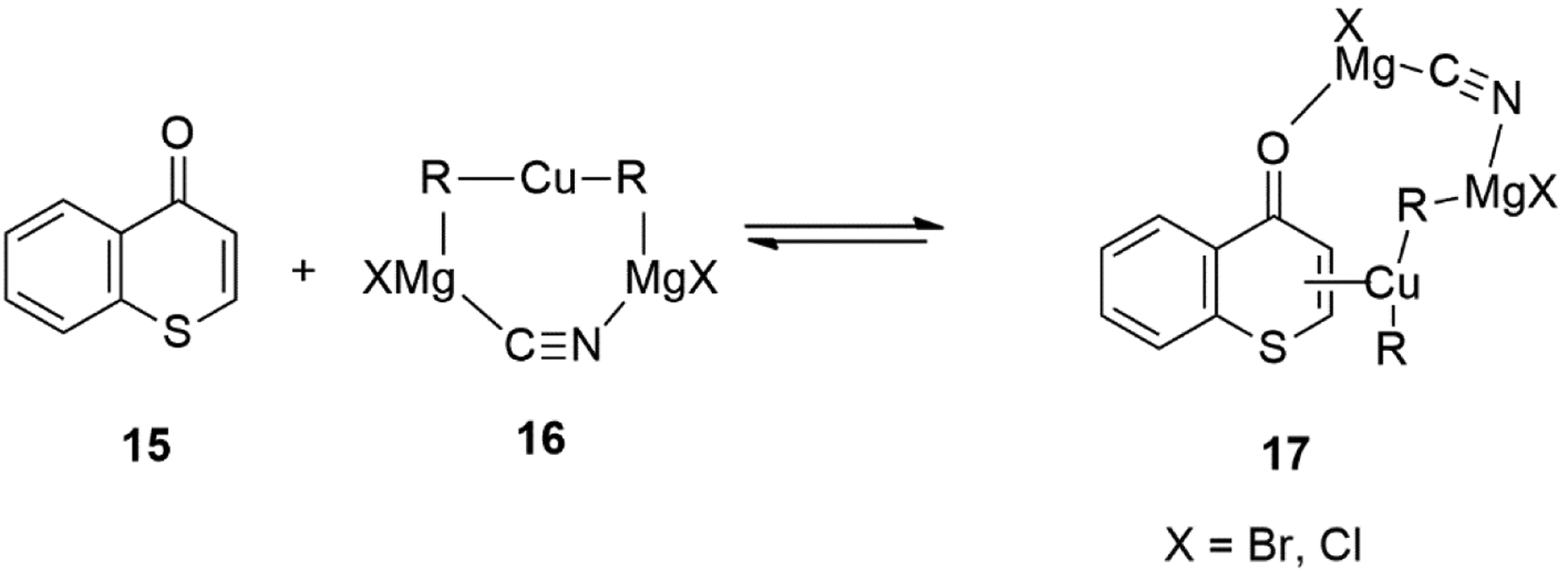
Plausible Reactive Intermediates in Cu-catalyzed Conjugate Addition to Thiochromones.

The plausible mechanism on the copper-catalyzed 1,4-conjugate addition reactions of Grignard reagents [i.e., RMgX, X = Br, Cl] with thiochromones **15** in THF would involve the formation of similar π-complexes **17** (Figure 15). The reactive species likely would be the heterodimeric structure of the contact ion-pair (CIP) **16** in THF (Figure 15).

**Figure 16.**
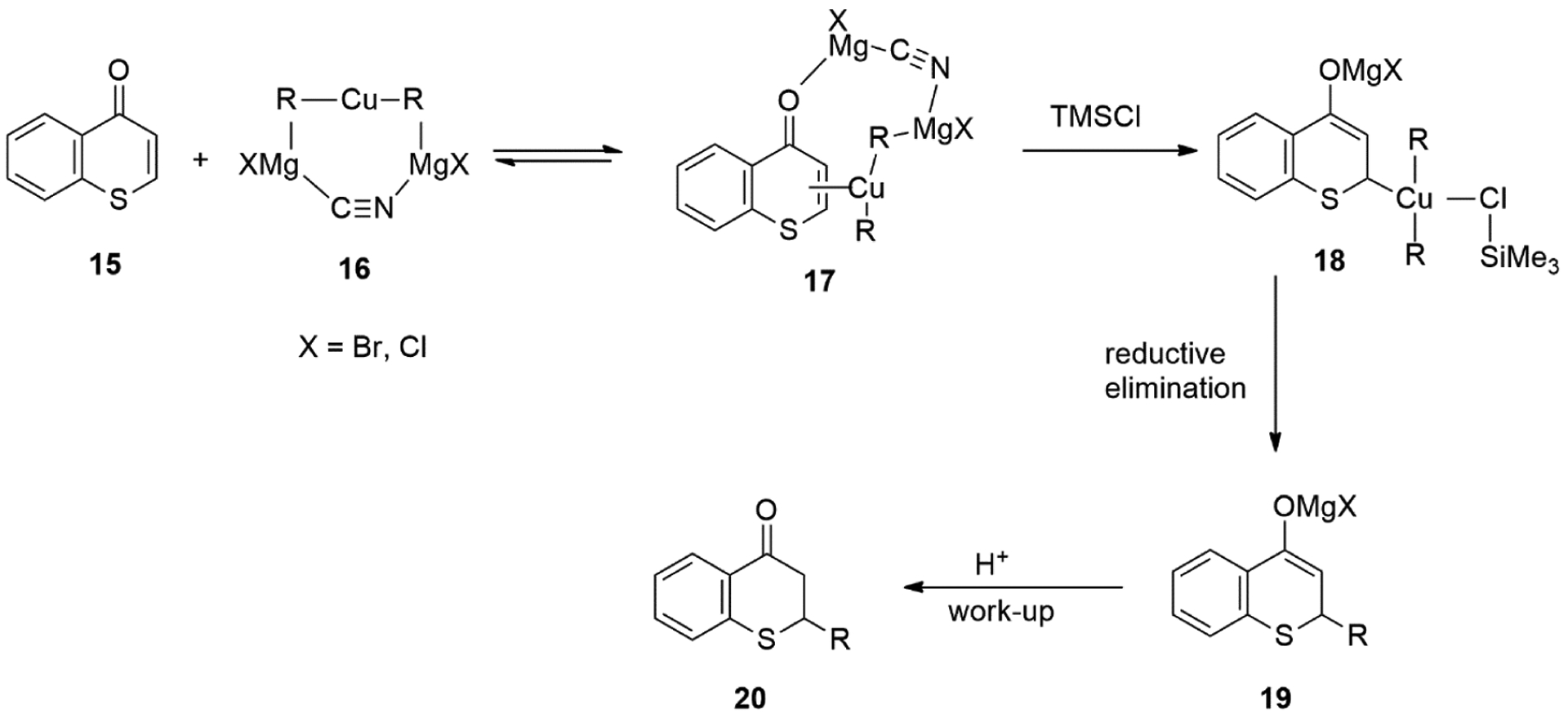
The Role of TMSCl in the Conjugate Addition of Grignard Reagents to Thiochromones.

Lewis acids such as TMSCl were reported to greatly accelerate the organocuprate reactions [63,64]. With the Cu-catalyzed 1,4-conjugate addition of Grignard reagents, it is likely that TMSCl stabilized the π-complex **17** by converting it to a reactive tetravalent copper species **18**, which is capable of a rapid reductive elimination to form 1,4-adduct (Figure 16).

## 6. Discussions—Challenges and Perspectives

Sulfur-heterocycles are much less studied compared to their corresponding oxygen counterparts. For example, the reactivities of thiochromones and the oxygen counterpart chromones are very different. Thiochromones are usually less reactive due to a higher degree of electron delocalization of lone pair on the sulfur atom, and this places higher electron density on the Michael acceptors and makes it less electron deficient comparing to oxygen counterparts, thus reducing the reactivity towards organometallic reagents. Recent advances in the synthetic approaches towards the successful synthesis of 2-substituted thiochroman-4-ones including 2-alkylthiochroman-4-ones, 2-arylthiochroman-4-ones (thioflavanones), 2-alkenyl thiochroman-4-ones, and 2-alkynyl thiochroman-4-ones all involved the use of Lewis acids such as TMSOTf, TMSCl, and TMSI [58–61]. The progress highlighted the importance of Lewis acids in activating thiochromones and, thus, increased reactivities. These recent advances further demonstrated the versatility of Cu-catalyzed reactions to include the 1,4-conjugate addition reactions to heterocyclic Michael acceptors such as thiochromones. The copper-catalyzed 1,4-conjugate addition of Grignard reagents are appealing due to the readily availability of Grignard reagents, the ease in preparation from corresponding halide compounds and the broad scope of Grignard reagents that will enable the synthesis of a large library of analogs for biological screening. The Cu-catalyzed conjugate addition of organometallic reagents offered a quick entry into an important class of sulfur-heterocycles, 2-subsituted thichroman-4-ones from common starting materials-thiochromones. The ability to add all three different carbon centers (sp, sp^2^, sp^3^) such as alkyl, aryl, alkenyl, and alkynyl groups are important and showcase the versatility of these transformations including copper-catalyzed 1,4-addition of Grignard reagents [58,59], the copper-catalyzed formal conjugate addition of alkynyl groups [60] as well as the addition of alkenyl Grignard reagents to thiochromone promoted by TMSOTf [61]. These recent advances on the 1,4-addition or formal 1,4-addition to thiochromones are important, as they enable a quick entry into large varieties of 2-substituted thiochroman-4-ones, an important class of S-heterocycles known for their biological activities. One of the challenges in the reaction of organocuprate reagents is that it often requires stoichiometric amount of copper salts. It would always be more desirable to develop 1,4-conjugate addition of organometallic reagents including Grignard reagents using catalytic amount of copper salts. There are still challenges in developing a more general Cu-catalyzed enantioselective 1,4-conjugate addition of alkyl Grignard reagents to thiochromones that will offer high enantioselectivity for a broader scope of both Grignard reagents as well as thiochromones. A broader scope of akenyl and alkynyl Grignard reagents that would lead to 2-akenyl, 2-alkynyl substituted thiochroman-4-ones with excellent enantioselectivities are also desirable.

## 7. Conclusions

Carbon–carbon bond formation is one of the most important transformations in the synthesis of carbon framework of complex molecules in organic synthesis. Among the many synthetic methodologies for carbon–carbon bond formation, organocopper reagents are one of the most reliable organometallic reagents for this purpose. The versatility of Cu-catalyzed reactions was demonstrated by their applications in a variety of synthetic transformations, including the 1,4-conjugate addition reactions. The Cu-catalyzed conjugate addition of organometallic reagents offered straightforward access to an important class of sulfur-heterocycles, 2-alkylthiochroman-4-ones and thioflavanones, from common starting materials-thiochromones. This paper provided a brief review on recent progress on the synthesis of an important class of sulfur-heterocycles-2-alkylthiochroman-4-ones and thioflavanones via the conjugate additions of Grignard reagents to thiochromones catalyzed by copper catalysts. Recent progress on the synthesis of 2-alkynyl thiochroman-4-ones via the Cu-catalyzed formal conjugate addition of alkynyl groups to thiochromones and 2-alkenylation thiochromones via 1,4-additions of alkenyl Grignard reagents promoted by TMSOTf were also covered in this review. These recent progresses on the addition of alkyl, aryl, and alkenyl to thiochromones via 1,4-conjugate addition of Grignard reagents as well as the formal conjugate addition of alkynyl groups to thiochromones catalyzed of Cu (I) salts provides a quick entry into 2-substituted thiochroman-4-ones, an important class of S-heterocycles known for their biological activities.

## References

[1] Breit B; Schmidt Y Directed reactions of organocopper reagents. Chem. Rev 2008, 108, 2928. 18698734 10.1021/cr078352c

[2] Taylor RJK (Ed.) Organocopper Reagents: A Practical Approach; Oxford University Press: Oxford, UK, 1994.

[3] Krause N (Ed.) Modern Organocopper Chemistry; John Wiley & Sons: New York, NY, USA, 2002.

[4] Krause N; Hoffmann-Röder A Recent advances in catalytic enantioselective Michael additions. Synthesis 2001, 2001, 171.

[5] Lipshutz BH Organometallics in Synthesis: A Manual; Schlosser M, Ed.; Chapter 4; John Wiley & Sons: Chichester, UK, 1994; p. 283.

[6] Krause N; Gerold A Regio- and Stereoselective Syntheses with Organocopper Reagents. Angew. Chem. Int. Ed. Engl 1997, 36, 186.

[7] Alexakis A; Benhaim C Enantioselective copper-catalysed conjugate addition. Eur. J. Org. Chem 2002, 2002, 3221. 10.1021/ol0059596 10990401

[8] Harutyunyan SR; Hartog TD; Geurts K; Minnaard AJ; Feringa BL Catalytic asymmetric conjugate addition and allylic alkylation with Grignard reagents. Chem. Rev 2008, 108, 2824. 18698733 10.1021/cr068424k

[9] Guo F; Jeffries MC; Graves BN; Graham SA; Pollard DA; Pang G; Chen HY A rapid entry into thioflavanones via conjugate additions of diarylcuprates to thiochromones. Tetrahedron 2017, 73, 5745–5750.

[10] Bass SA; Parker DM; Bellinger TJ; Eaton AS; Dibble AS; Koroma KL; Sekyi SA; Pollard DA; Guo F Development of Conjugate Addition of Lithium Dialkylcuprates to Thiochromones: Synthesis of 2-Alkylthiochroman-4-ones and Additional Synthetic Applications. Molecules 2018, 23, 1728. 30011953 10.3390/molecules23071728 PMC6099951

[11] In Lee J Synthetic Approaches to 2-Alkylthiochroman-4-ones and Thioflavanones. Bull. Korean Chem. Soc 2021, 42, 852–862.

[12] Damani LA (Ed.) Sulphur-Containing Drugs and Related Organic Compounds; Wiley: New York, NY, USA, 1989.

[13] Clayden J; MacLellan P Asymmetric synthesis of tertiary thiols and thioethers. Beilstein J. Org. Chem 2011, 7, 582–595. 21647256 10.3762/bjoc.7.68 PMC3107483

[14] Ingall AH Thiopyrans and Fused Thiopyrans. In Comprehensive Heterocyclic Chemistry; Katritzky AR, Rees CW, Eds.; Pergamon Press: Oxford, UK, 1984; Volume 3, p. 885.

[15] Schneller SW Thiochromanones and Related Compounds. In Advances in Heterocyclic Chemistry; Katritzky AR, Boulton AJ, Eds.; Academic Press: New York, NY, USA, 1975; Volume 18, p. 59.

[16] Acton AQ (Ed.) Sulfur Compounds: Advances in Research and Application; Scholarly Editions: Atlanta, GA, USA, 2012.

[17] Nielsen SFE; Nielsen O; Olsen GM; Liljefors T; Peters D Novel Potent Novel Potent Ligands for the Central Nicotinic Acetylcholine Receptor: Synthesis, Receptor Binding, and 3D-QSAR Analysis. J. Med. Chem 2000, 43, 2217–2226. 10841800 10.1021/jm990973d

[18] Smith BR; Eastman CM; Njardarson JT Beyond C, H, O and N! Analysis of the Elemental Composition of U.S. FDA Approved Drug Architectures. J. Med. Chem 2014, 57, 9764–9773. 25255063 10.1021/jm501105n

[19] Takimiya K; Osaka I; Mori T; Nakano M Organic Semiconductors Based on [1]Benzothieno[3,2-*b*][1]benzothiophene Substructure. Acc. Chem. Res 2014, 47, 1493–1502. 24785263 10.1021/ar400282g

[20] Joyce NI; Eady CC; Silcock P; Perry NB; Van Klink JW Fast Phenotyping of LFS-Silenced (Tearless) Onions by Desorption Electrospray Ionization Mass Spectrometry (DESI-MS). J. Agric. Food Chem 2013, 61, 1449–1456. 23350988 10.1021/jf304444s

[21] Mishra A; Ma CQ; Bauerle P Functional Oligothiophenes: Molecular Design for Multidimensional Nanoarchitectures and Their Applications. Chem. Rev 2009, 109, 1141–1276. 19209939 10.1021/cr8004229

[22] Lin DY; Zhang SZ; Block E; Katz LC Encoding social signals in the mouse main olfactory bulb. Nature 2005, 434, 470–477. 15724148 10.1038/nature03414

[23] Wermuch CG Molecular variations based on isosteric replacements. In The Practice of Medicinal Chemistry; Wermuth CG, Ed.; Academic Press: San Diego, CA, USA, 1996; pp. 203–237.

[24] Glaser C Ber. 2, 422 (1869); Glaser C Liebigs Ann. Chem 1870, 154, 159.

[25] Ullmann F Ber. deut. Chem. Ges. 29, 1878 (1896). Liebigs Ann. Chem 1904, 332, 38.

[26] Ullmann F; Sponagel P Ueber die phenylirung von phenolen. Chem. Ber 1905, 38, 2211–2212.

[27] Reich R Nouveaux composés organométalliques: Le cuivre phényle et l’argent phényle. Compt. Rend 1923, 177, 322.

[28] Gilman H; Straley JM Relative reactivities of organometallic compounds. XIII. Copper and silver. Rec. Trav. Chim 1936, 55, 821.

[29] Kharasch MS; Tawney PO Factors determining the course and mechanisms of Grignard reactions. II. The effect of metallic compounds on the reaction between isophorone and methylmagnesium bromide. J. Am. Chem. Soc 1941, 63, 2308–2316.

[30] Gilman H; Jones RG; Woods LAJ The preparation of methylcopper and some observations on the decomposition of organocopper compounds. Org. Chem 1952, 17, 1630.

[31] House HO; Respess WL; Whitesides GMJ The chemistry of carbanions. XII. The role of copper in the conjugate addition of organometallic reagents^1^. Org. Chem 1966, 31, 3128.

[32] Lipshutz BH; Sengupta S Organocopper reagents: Substitution, conjugate addition, carbo/metallocupration, and other reactions. Org. React 1992, 41, 135.

[33] Choi EJ; Lee JI; Kim G-H Evaluation of the anticancer activities of thioflavanone and thioflavone in human breast cancer cell lines. Int. J. Mol. Med 2012, 29, 252–256. 22076075 10.3892/ijmm.2011.834

[34] Vargas E; Echeverri F; Vélez ID; Robledo SM; Quiňones W Synthesis and Evaluation of Thiochroman-4-one Derivatives as Potential Leishmanicidal Agents. Molecules 2017, 22, 2041. 29186046 10.3390/molecules22122041 PMC6149949

[35] Zhao J; Li H-Z; Suo H; Wang Y; Yang C; Ma Z; Liu Y Cytoxic effect of three novel thiochromanone derivatives on tumor cell in vitro and underlying mechanism. Glob. Adv. Res. J. Med. Sci 2014, 3, 240–250.

[36] Harborne JB The Flavonoids: Advances in Research Since 1980; Chapman and Hall: New York, NY, USA, 1988.

[37] Harborne JB; Williams CA Anthocyanins and other flavonoids. Nat. Prod. Rep 1995, 12, 639–657. 10.1039/b006257j 11476484

[38] Pietta PG Flavonoids as antioxidants. J. Nat. Prod 2000, 63, 1035–1042. 10924197 10.1021/np9904509

[39] Andersen OM; Markham KR (Eds.) Flavonoids: Chemistry, Biochemistry and Applications; Taylor & Francis: London, UK, 2006.

[40] Philipp A; Jirkovsky I; Martel RR Synthesis and antiallergic properties of some 4*H*, 5*H*-pyrano[3,2-*c*][1]benzopyran-4-one, 4*H*,5*H*-[1]benzothiopyrano[4,3-*b*]pyran-4-one, and 1,4-dihydro-5*H*-[1]benzothiopyrano[4,3-*b*]pyridine-4-one derivatives. J. Med. Chem 1980, 23, 1372–1376. 7452691 10.1021/jm00186a016

[41] Ramalingam K; Thyvelikakath GX; Berlin KD; Chesnut RW; Brown RA; Durham NN; Ealick SE; Van der Helm D Synthesis and biological activity of some derivatives of thiochroman-4-one and tetrahydrothiapyran-4-one. J. Med. Chem 1977, 20, 847–850. 406397 10.1021/jm00216a024

[42] Wang HK; Bastow KF; Cosentino LM; Lee KH Antitumor Agents. 166. Synthesis and Biological Evaluation of 5,6,7,8-Substituted-2-phenylthiochromen-4-ones. J. Med. Chem 1996, 39, 1975–1980. 8642556 10.1021/jm960008c

[43] Holshouser MH; Loeffler LJ; Hall IH Synthesis of peptides by the solid-phase method. 6. Neurotensin, fragments, and analogs. J. Med. Chem 1981, 24, 853–858. 7265123 10.1021/jm00136a004

[44] Dhanak D; Keenan RM; Burton G; Kaura A; Darcy MG; Shah DH; Ridgers LH; Breen A; Lavery P; Tew DG; Benzothiopyran-4-one based reversible inhibitors of the human cytomegalovirus (HCMV) protease. Bioorg. Med. Chem. Lett 1998, 8, 3677–3682. 9934494 10.1016/s0960-894x(98)00666-0

[45] Nussbaumer P; Lehr P; Billich A 2-Substituted 4-(Thio)chromenone 6-O-Sulfamates: Potent Inhibitors of Human Steroid Sulfatase. J. Med. Chem 2002, 45, 4310–4320. 12213072 10.1021/jm020878w

[46] Kataoka T; Watanabe S; Mori E; Kadomoto R; Tanimura S; Kohno M Synthesis and structure-activity relationships of thioflavone derivative as specific inhibitors of the ERK-MAP kinase signaling pathway. Bioorg. Med. Chem 2004, 12, 2397–2407. 15080936 10.1016/j.bmc.2004.02.002

[47] Soni DV; Jacobberger JW Gene modulation by Cox-1 and Cox-2 specific inhibitors in human colorectal carcinoma cancer cells. Cell Cycle 2004, 3, 349–357. 14633654 10.1093/carcin/bgh016

[48] Bondock S; Metwally MA Thiochroman-4-ones: Synthesis and reactions. J. Sulfur Chem 2008, 29, 623–653.

[49] Dalla Via L; Marciani Magno S; Gia O; Marini AM; Da Settimo F; Salerno S; La Motta C; Simorini F; Taliani S; Lavecchia A; Benzothiopyranoindole-Based Antiproliferative Agents: Synthesis, Cytoxicity, Nucleic Acids Interaction, and Topoisomerases Inbibition Properties. J. Med. Chem 2009, 52, 5429–5441. 19725581 10.1021/jm900627v

[50] Bates DK; Li K Stannous Chloride-Mediated Reductive Cyclization-Rearrangement of Nitroarenyl Ketones. J. Org. Chem 2002, 67, 8662–8665. 12444654 10.1021/jo0259921

[51] Aramaki Y; Seto M; Okawa T; Oda T; Kanzaki N; Shiraishi M Synthesis of 1-Benzothiepine and 1-Benzazepine Derivatives as Orally Active CCR5 Antagonists. Chem. Pharm. Bull 2004, 52, 254–258. 10.1248/cpb.52.254 14758013

[52] Dike SY; Ner DH; Kumar A A New Enantioselective Chemoenzymatic Synthesis of R-(−)Thiazesim Hydrochloride. Bioorg. Med. Chem. Lett 1991, 1, 383–386.

[53] Phippen CBW; McErlean CSPA 1,5-Benzothiazepine Synthesis. Tetrahedron Lett. 2011, 52, 1490–1492.

[54] Li W; Schlepphorst C; Daniliuc C; Glorius F Asymmetric Hydrogenation of Vinylthioethers: Access to Optically Active 1,5-Benzothiazepine Derivatives. Angew. Chem. Int. Ed 2016, 55, 3300–3303. 10.1002/anie.201512032 26846168

[55] Fang X; Li J; Wang CJ Organocatalytic Asymmetric Sulfa-Michael Addition of Thiols to α,β-Unsaturated Hexafluoroisopropyl Esters: Expeditious Access to (R)-Thiazesim. Org. Lett 2013, 15, 3448–3451. 23772965 10.1021/ol4015305

[56] Fukata Y; Asano K; Matsubara S Facile Net Cycloaddition Approach to Optically Active 1,5-Benzothiazepines. J. Am. Chem. Soc 2015, 137, 5320–5323. 25856510 10.1021/jacs.5b02537

[57] Ortiz P; Lanza F; Harutyunyan SR 1,2- Versus 1,4-Asymmetric Addition of Grignard Reagents to Carbonyl Compounds. In Progress in Enantioselective Cu(I)-catalyzed Formation of Stereogenic Centers; Harutyunyan SR, Ed.; Springer Press: Basel, Switzerland, 2016; pp. 99–134.

[58] Bellinger TJ; Harvin T; Pickens-Flynn T; Austin N; Whitaker SH; Tang Yuk Tutein MLC; Hukins DT; Deese N; Guo F Conjugate Addition of Grignard Reagents to Thiochromones Catalyzed by Copper Salts: A Unified Approach to Both 2-Alkylthiochroman-4-One and Thioflavanone. Molecules 2020, 25, 2128. 32370080 10.3390/molecules25092128 PMC7248974

[59] Luo S; Meng L; Yang Q; Wang J Cu-Catalyzed Conjugate Addition of Grignard Reagents to Thiochromones: An Enantioselective Pathway for Accessing 2-Alkylthiochromanones. Synlett 2018, 29, 2071–2075.

[60] Meng L; Ngai KY; Chang X; Lin Z; Wang J Cu(I)-Catalyzed Enantioselective Alkynylation of Thiochromones. Org. Lett 2020, 22, 1155–1159. 31961693 10.1021/acs.orglett.0c00005

[61] Hoettecke N; Rotzoll S; Albrecht U; Lalk M; Fischer C; Langer P Synthesis and antimicrobial activity of 2-alkenylchroman-4-ones, 2-alkenylthiochroman-4-ones and 2-alkenylquinol-4-ones. Bioorg. Med. Chem 2008, 16, 10319–10325. 18977661 10.1016/j.bmc.2008.10.043

[62] Bertz SH; Cope S; Murphy M; Ogle CA; Taylor BJ Rapid injection NMR in mechanistic organocopper chemistry. Preparation of the elusive copper (III) intermediate. J. Am. Chem. Soc 2007, 129, 7208–7209. 17506552 10.1021/ja067533d

[63] Corey EJ; Boaz NW Evidence for a reversible d, π-complexation, β-cupration sequence in the conjugate addition reaction of Gilman reagents with α, β-enones. Tetrahedron Lett. 1985, 26, 6015–6018.

[64] Bertz SH; Miao G; Rossiter BE; Snyder JP Effect of TMSCl on the Conjugate Addition of Organocuprates to α-enones: A New Mechanism. J. Am. Chem. Soc 1995, 117, 11023–11024.

